# Modification of the Superomedial Pedicle in Wise-Pattern Breast Reduction: New Sling Suspension Technique to Prevent (Pseudo) Ptosis

**DOI:** 10.29252/wjps.8.3.305

**Published:** 2019-09

**Authors:** Martain Loonen, Adnan Tahir

**Affiliations:** King’s College Hospital London, Adnan Tahir, UAE

**Keywords:** Superomedial pedicle, Wise-pattern, Breast reduction, Ptosis

## Abstract

**BACKGROUND:**

The combination of the superomedial pedicle with the traditional Wise-pattern skin resection has gained increasing popularity for its versatility and ability to achieve significant reduction of breast parenchyma and skin envelope.

**METHODS:**

The author describes a reproducible new dermal suspension technique for cranial stabilization and fixation of the superomedial pedicle in Wise inverted T breast reductions to prevent pseudoptosis.

**RESULTS:**

One patient had a small dermal skin abscess caudal from the right areola as a tissue reaction on the remaining suture. The other peri- and post-operative cases were uncomplicated. Two patients experienced a two-week postoperative pain in the areas treated by liposuction. There was no reduced or increased sensibility of the nipple-areola complex. No signs of (pseudo) ptosis were seen. There was a 100% patient satisfaction rate.

**CONCLUSION:**

Our technique denoted to a direct support of the pedicle using a non-interrupted dermal suspension flap continued with the pedicle to be a medial, caudal and lateral support for the pedicle. The dermal sling reduced the anterior force generated by the pedicle. The pedicle enfolded by the dermal flap formed a vital basis for ingrowth in the surrounding tissue.

## INTRODUCTION

The combination of the superomedial pedicle with the traditional Wise-pattern skin resection has gained increasing popularity for its versatility and ability to achieve significant reduction of breast parenchyma and skin envelope with improved contour and lasting results. The superomedial pedicle brings improved upper pole fullness and breast shape, while the Wise-pattern skin resection allows for its reproducible, standardized markings and elimination of excess skin in both the vertical and horizontal dimensions.^1^ Pseudoptotis has been reported in inverted T-skin excisions especially when the inferior pedicle technique is used.^2^ The superomedial pedicle may reduce the risk of (pseudoptosis), nevertheless, the weight of the pedicle is only supported by the superior skin flaps and breast parenchyma in case no additional suspension techniques are used. Recurrent breast ptosis may occur especially in patients with poor skin quality. The author describes a new dermal suspension technique for cranial stabilization and fixation of the superomedial pedicle in Wise pattern inverted T breast reductions.

## MATERIAL AND METHODS

**Fig. 1:**
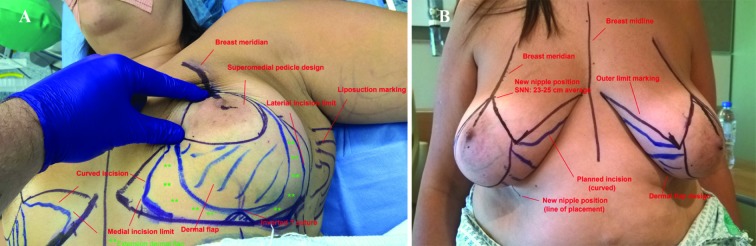
Preoperative markings. Note that the dermal flap design can be extended medially and laterally (A and B)

Figure 1 shows inframammary fold marking including the final medial and lateral inframammary fold incision is approximately 2 cm shorter than the total inframmary fold length, breast meridian marking, and new nipple position consisted of the new nipple position is at the most projecting part of the breast, most likely at the inframammary fold. The nipple position should not be higher than the IMF in case of a concave upper pole of the breast. The average suprasternal notch-nipple distance is more caudal in the ptotic breast with reduced upper pole volume. This prevents excessive resection of breast parenchyma with difficulties in natural shaping of the breasts. In addition, this prevents the nipples being placed too high (too cranial).

Regarding the areola opening, the final position of the nipple-areola position is made intraoperatively with the patient in a sitting position. This prevents the nipple-areola complex being placed too high (too cranial) compared with a preoperative planned nipple position. Regarding the skin resection pattern which was the author preference, a 7 cm point from the new nipple position in the direction of the inframmary fold was marked. A point was marked 5.5 cm medial and lateral from this point. The medial and lateral vertical limb was drawn connecting these dots. 

The final vertical limbs were approximately 8.5 cm in length with a base of 11 cm. The caudal ends of the limbs were approximated with the surgeon hands to simulate the reduction. The nipple position and base width were adjusted based on this maneuver. The final inframammary incisions were planned a few centimeters shorter than the preexisting inframammary fold length, as long as care was taken to resect the breast tissue that would otherwise remain in that area. The suture for the inverted T marking was placed cranially on a “hill top “position to prevent extensive traction on the inverted T suture. The medial and lateral incisions were curved to extent the length of the medial and lateral incisions which provided additional tension reduction on the inverted T suture (Figure 1B). 

Additional liposuction of the lateral breast and axilla area was performed in case of lipoaccumulation in these anatomical areas. The liposuction procedure reduced the length of the lateral inframmary fold incision. The lateral marking of the inframammary fold was 2 cm shorter than the actual inframammary fold as described above. 

In operative technique, the patient was in supine position. Subcutaneous infiltration was undertaken with a solution of 500 mL 0.9% NaCl, 25 mL 1%lidocaine, 6 mL 8.4% sodium bicarbonate (1 mEq/mL) and 0.5 mL 1:1000 adrenaline at the skin resection patterns and the liposuction areas, but not in the pedicle area (author preference). Nipple-areola complex marking was conducted with a diameter of 4.5 cm. Deepithelialization of the superomedial pedicle was done with preservation of the nipple-areola complex. Then incision of the skin resection lines was performed and prepectoral fascia dissection was carried out in a Lotus flap pattern without dissection at the medial side of the pedicle.

**Fig. 2:**
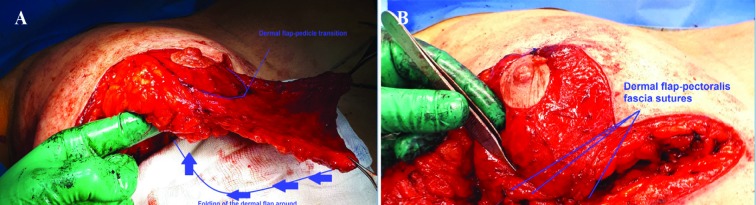
Perioperative. The dermal flap was folded around the pedicle. The dermal flap was sutured to the pectoralis fascia with non-absorbable sutures (A and B).

Marking of the hammock suspension flap pattern was designed on the skin and later, the deepithelialization and preparation of an adipodermal suspension flap. The excessive glandular-adipose tissue was excised below the suspension flap and the superomedial pedicle was dissected. The medial and lateral adipocutaneous-glandular excision was undertaken according to the Wise pattern and folding of the suspension flap was done around the pedicle to form a hammock (Figure 2A). 

The patient was in half sitting position for approximation of the inverted T adipocutaneous flaps. Positioning of the pedicle was central on the thoracic wall until the desired breast shape was reached. Patient back was in supine position and the fixation of the dermal sling to the pectoralis fascia was with non-absorbable Prolene® 3-0 sutures (Figure 2B). Fixation of the inverted T adipocutaneous flaps to the pectoralis fascia was with Prolene® 2-0 suture. Scarpa fascia-pectoralis fascia sutures was carried out using Prolene® 3-0 at the level of the inframammary fold. Liposuction was at the lateral and axillary areas. Deep dermal sutures were conducted using PDS® 3-0 and 4-0. Intradermal sutures were with Monocryl® 4-0. 

Patient in half sitting position was marked in the desired nipple position. Marking of the new nipple-areola complex was possible with a circular diameter of 4.5 cm. The medial deepithelialization of the new circular areola and full thickness excision of the lateral circular area were further undertaken. Cranial transposition of the pedicle and the nipple into the circular defect was carried out and circular deep dermal area was sutured with PDS® 4/0 and with an intradermal suture of Monocryl® 5-0 and using 3M Steri-Strips®, and Fucidin® intertulle. The gauze and Tegaderm® were applied with pad dressings at the sutured areas with an opening at the nipple-areola complex to evaluate the vascularity. Fixation of the breasts was performed with supportive 3M Microfoam® tape.

## RESULTS

**Fig. 3:**
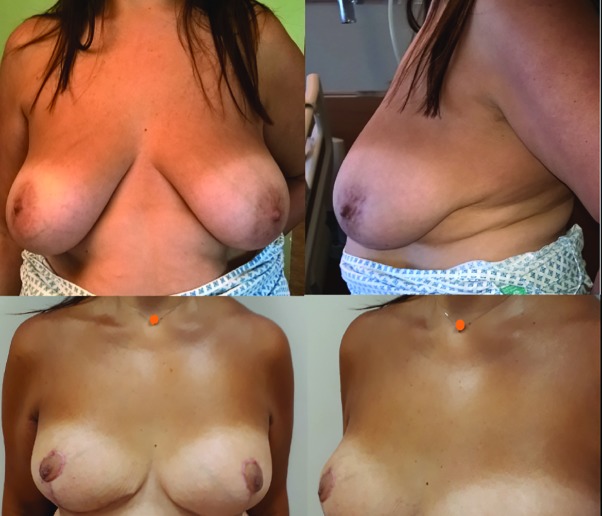
Pre- and postoperative evaluation, patient 1. A 32-year-old patient with prominent breast asymmetry and volume difference. Three months postoperative. Some breast edema was still present at the left breast. Patient had a small dermal skin abscess caudal from the right areola as a tissue reaction on the remaining suture

**Fig. 4:**
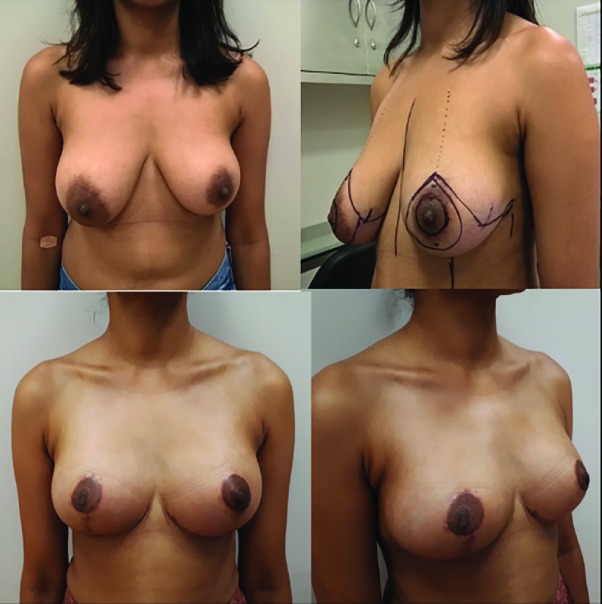
Pre-and postoperative evaluation, patient 2. A 51-year-old obese patient with prominent breast ptosis and asymmetry. Four months postoperative. Note the small medial dog ears which were retracted in most cases within 6 months after the surgery. The patient did not request any additional corrections

We used the dermal suspension technique of the superomedial pedicle technique in 3 surgical cases (Figure 3 and 4). All dermal suspension flaps and pedicles were vital. One patient had a small dermal skin abscess caudal from the right areola as a tissue reaction on the remaining suture. The other peri- and post-operative cases were uncomplicated. The dressings and supportive foam tape were removed 5 days postoperative. Postoperative histopathology evaluation of the resected breast tissue was done for all the operated cases. 

Two patients experienced a two-week postoperative pain in the areas treated by liposuction. There was no reduced or increased sensibility of the nipple-areola complex. A supportive breast garment was advised for 6 weeks. The mean follow up time was 3 months. No signs of (pseudo) ptosis were seen. There was a 100% patient satisfaction rate and all patients were happy to recommend the procedure. One patient only had a telephone consultation follow up as she left the country without a postoperative picture.

## DISCUSSION

Ptosis of the breasts in the postoperative phase after breast reduction which was associated with caudal displacement of the breast tissue below the inframammary fold (bottoming out) with a prominent cranial appearance of the nipple-areola complex on the breast. This would lead to unsatisfactory cosmetic results. The development of (pseudo) ptosis is explained by the skin only support of the adipose-glandular tissue after a Wise pattern breast reduction.

Additional techniques to support the adipose-glandular tissue and to prevent further postoperative ptosis consist of (i) Scarpa fascia sutures to the pectoralis fascia at the inframammary fold, (ii) Fixation of the inverted T point to the pectoralis fascia or rib periosteum. The latter technique is associated with more postoperative pain, (iii) Dermal straps to the pectoralis muscle,^3^ (iv) Glandular-subcutaneous sutures,^4^ (v) Pedicle suspension and plication in the pectoral fascia,^5^ (vi) Circular non-absorbable dermal sutures with glandular shaping,^6^ (vii) Biocompatible or non-biocompatible mesh to replace the supporting function of the ligamentous suspension,^7^^,^^8^ and (viii) Dermal suspension flaps.

Bottoming out has been described in the literature, especially for the inferior pedicle technique. By using the inferior pedicle, the surgeon relies on the skin envelope to hold up the weight of the breast tissue and pedicle. Many surgeons create a short, tight lower pole to help prevent early bottoming-out.^1^ A prominent superomedial pedicle in a Wise pattern breast reduction forms a prominent weight on the surrounding superior skin and breast tissue. The first 2 techniques described above are based on skin support only of the pedicle. Pedicles with a substantial amount of adipose tissue may lack supportive tissue to be fixated to the pectoralis fascia with gradual loss of support. 

Dermal flaps form an additional supportive structure for the adipose-glandular tissue. Dermal suspension flaps in breast reduction have been described for the inferior pedicle technique and for the superomedial pedicle technique.^9^^,^^10^ A laterally dermal fascial flap has been described flap for the superomedial pedicle, independent from the pedicle with caudal support of the pedicle.^10^ The lateral dermal fascial flap technique showed higher patient satisfaction for patients who underwent dermo-fascial suspension to the chest wall compared with a classic superomedial pedicle without suspension. Significant differences in terms of suprasternal notch-nipple and nipple-inframammary fold distances were seen among both groups.^10^

Our technique describes a direct support of the pedicle using a non interrupted dermal suspension flap continued with the pedicle to be a medial, caudal and lateral support for the pedicle. The dermal sling reduced the anterior force generated by the pedicle. The pedicle enfolded by the dermal flap formed a vital basis for ingrowth in the surrounding tissue. Our study was limited to 3 surgical cases. An additional analysis between a control group (without the dermal suspension) and the dermal suspension group is necessary to evaluate potential significant statistical differences. In addition, a longer follow up time is necessary to evaluate the long-term effects as our study had an average follow up of 3 months. The hammock dermal suspension for the superomedial pedicle in a Wise pattern breast reduction was shown to be a reproducible and promising technique to prevent (pseudo) ptosis of the operated breasts.

## CONFLICT OF INTEREST

The authors declare no conflict of interest.

## References

[1] Brown RH, Siy R, Khan K, Izaddoost S (2015). The Superomedial Pedicle Wise-Pattern Breast Reduction: Reproducible, Reliable, and Resilient. Semin Plast Surg.

[2] Spear SL, Howard MA (2003). Evolution of the vertical reduction mammaplasty. Plast Reconstr Surg.

[3] Hinderer UT (1976). The dermal brassiere mammaplasty. Clin Plast Surg.

[4] Lassus C (1996). A 30-year experience with vertical mammaplasty. Plast Reconstr Surg.

[5] Pennington DG (2006). Improving the results of inferior pedicle breast reduction using pedicle suspension and plication. Aesthetic Plast Surg.

[6] Benelli L (1990). A new periareolar mammaplasty: the “round block” technique. Aesthetic Plast Surg.

[7] van Deventer PV, Graewe FR, Wuringer E (2012). Improving the longevity and results of mastopexy and breast reduction procedures: reconstructing an internal breast support system with biocompatible mesh to replace the supporting function of the ligamentous suspension. Aesthetic Plast Surg.

[8] Bustos RA (1992). Periareolar mammaplasty with silicone supporting lamina. Plast Reconstr Surg.

[9] Kankaya Y, Oruc M, Sungur N, Aslan OC, Gursoy K, Ozer K, Kocer U (2016). Four flap suspension technique for prevention of bottoming out after breast reduction. Ann Surg Treat Res.

[10] Abdelaal MM, Aboelatta YA (2015). Dermo-Fascial Suspension for Better Contouring and Long Lasting Results in Reduction Mammoplasty. Aesthetic Plast Surg.

